# Quantifying variability of mitochondrial markers in m3243A > G myopathy

**DOI:** 10.1038/s41598-025-33106-3

**Published:** 2025-12-19

**Authors:** Tiago M. Bernardino Gomes, Jordan B. Childs, Valeria Di Leo, Charlotte Warren, Gavin Hudson, Doug M. Turnbull, Conor Lawless, Amy E. Vincent

**Affiliations:** 1 Mitochondrial Research Group, Translational and Clinical Research Institute, Faculty of Medical Sciences, Newcastle University, Newcastle upon Tyne, UK; 2 NHS Highly Specialised Service for Rare Mitochondrial Disorders of Adults and Children, Directorate of Neurosciences, Newcastle upon Tyne Hospitals NHS Foundation Trust, Newcastle upon Tyne, UK; 3 NIHR Biomedical Research Centre, Translational and Clinical Research Institute, Faculty of Medical Sciences, Newcastle University, Newcastle upon Tyne, UK; 4 Mitochondrial Research Group, Biosciences Institute, Newcastle University, Newcastle upon Tyne, UK

**Keywords:** Mitochondria, M.3243A > G, Myopathy, Oxidative phosphorylation, Heteroplasmy, Mitochondrial DNA copy number, Biological techniques, Biomarkers, Diseases, Genetics, Medical research, Physiology

## Abstract

Myopathy is a prevalent and disabling feature of mitochondrial disease, in which skeletal muscle accumulates fibres with mitochondrial dysfunction in a variable mosaic pattern. This intra-individual spatial heterogeneity, a key consideration in longitudinal assessments, remains largely uncharacterised, hindering mechanistic studies and clinical trials by obscuring or confounding findings. We quantified this variability in m.3243 A > G-related myopathy, a leading cause of adult mitochondrial disease. Post-mortem biopsies from *quadriceps femoris* and *tibialis anterior* muscles of four patients were analysed for single-fibre deficiency in oxidative phosphorylation (OXPHOS) complex I and IV, while homogenate mitochondrial DNA (mtDNA) copy number and m.3243 A > G heteroplasmy were respectively determined by quantitative PCR and pyrosequencing. Bootstrapped combinatorial analyses established thresholds for minimum meaningful change above the 97.5th percentile, while accounting for anatomical biopsy distancing. Spatial variability in the proportion of OXPHOS-deficient fibres increased with distancing; within the same muscle, this threshold was 13.8% for NDUFB8 and 9.8% for MT-CO1. Variability in mtDNA copy number modestly increased with distance, while m.3243 A > G heteroplasmy remained largely stable, with within-muscle thresholds of 1,136 copies per nucleus and 8.2%, respectively. These findings provide assay-specific thresholds and offer mechanistic and translational insights for trial design, patient monitoring, and reliable detection of disease progression or therapeutic response.

**Supplementary Information:**

The online version contains supplementary material available at 10.1038/s41598-025-33106-3.

## Introduction

Myopathy is a common feature of mitochondrial disease, characterised by progressive weakness, fatigue, and exercise intolerance^1–6^. The mitochondrial DNA (mtDNA) mutation m.3243 A > G is the leading cause of mitochondrial disease, with an estimated adult prevalence of 3.5–5.7 per 100,000^7–9^, representing up to around half of all adult mitochondrial cases^7,10^. The m.3243 A > G mutation manifests as a broad multisystemic disease^4–6,11–16^; however, myopathy is reported in 25–89% of cases^4–6,11–16^, and muscle-related symptoms substantially contribute to patient disability, irrespective of central nervous system involvement^1–4,6,16^. Thus, our poor understanding of the pathophysiology of m.3243 A > G-related myopathy has hindered therapeutic development and has left patients without effective treatments^17^.

Individual patient muscle cells, known as fibres, exhibit variable proportions of mutated and wild-type mtDNA molecules, known as heteroplasmy^18,19^. The phenotype is recessive, where a cell-specific heteroplasmy threshold must be reached to impair oxidative phosphorylation (OXPHOS) function and manifest disease^19–21^. Over time, mitotic mtDNA segregation, coupled with ongoing post-mitotic replication and degradation, drives and sustains fibre-to-fibre shifts in m.3243 A > G heteroplasmy^20,21^. Alongside other factors of mitochondrial dynamics, this heteroplasmy shift produces the mosaic pattern of OXPHOS-deficient and normal fibres that is characteristic of mitochondrial myopathy^19–21^.

Since 1987^22^, research has relied on heteroplasmy levels and OXPHOS function^22–25^ to characterise mitochondrial myopathy using muscle biopsies^19,26–28^. Repeat muscle biopsies are often used to monitor mitochondrial pathological changes over time^22,29–34^, particularly in disease progression studies and clinical trials^25,35–37^. However, despite strong phenotypical implications^21^, the intra-individual variability in muscle pathology has never been systematically quantified or consistently addressed by previous studies. Additionally, technical variability, especially when assessing OXPHOS, remained uncharacterised. The absence of variability thresholds to guide re-sampling strategies and data interpretation limits our ability to accurately distinguish disease progression or treatment effects from anatomical variability or assay noise. This gap has also hindered our understanding of the pathological mechanisms underlying m.3243 A > G-related myopathy, further delaying the development of targeted, disease-modifying therapies.

This study aimed to characterise intra-individual variability in mutation heteroplasmy, mtDNA copy number, and OXPHOS within the skeletal muscle of patients with m.3243 A > G-related myopathy. Our primary objective was to define assay-specific thresholds and provide methodological guidelines to account for intra-individual variability in longitudinal studies of disease mechanisms, biomarkers, and clinical trials, ultimately aiming to facilitate the translation of reliable findings into patient care. In parallel, by investigating the anatomical distribution of molecular pathology, we sought to generate mechanistic insights to improve interpretation of patient muscle phenotypes. To achieve these objectives, we systematically analysed multiple post-mortem biopsies of *quadriceps femoris* (QD) and *tibialis anterior* (TA) muscles from affected individuals. In doing so, we identified both biological and technical sources of variation affecting widely used quantification methods, particularly for OXPHOS. We developed a novel single-fibre OXPHOS classification method that effectively mitigates technical variability and enables detection of biologically meaningful changes. Although focused on the most common cause of adult mitochondrial myopathy, this work addresses broader knowledge gaps relevant to primary mitochondrial diseases and disorders involving secondary mitochondrial dysfunction^37,38^, and offers a generalisable experimental framework applicable across tissues, assays, and disease contexts.

## Materials and methods

### Skeletal muscle tissue biopsies

A combined total of 31 post-mortem biopsies from *quadriceps femoris* (QD) and *tibialis anterior* (TA) muscles across four patients with confirmed m.3243 A > G-related myopathy (Table 1) was obtained from the Newcastle Brain Tissue Resource (https://nbtr.ncl.ac.uk/, REC Ref: 19/NE/0008). Tissue sections from *biceps femoris* muscle biopsies, collected during *anterior cruciate* ligament surgery from individuals without known muscle disease, were provided by the Newcastle Biobank (REC Ref: 17/NE/0361) as controls for oxidative phosphorylation (OXPHOS) analysis. Control DNA for heteroplasmy assays was provided by the Newcastle Mitochondrial Research Biobank (REC Ref: 16/NE/0267). All samples, along with minimal demographic and clinical details, were supplied in anonymised form. Using biobank post-mortem tissues enabled us to obtain the multiple samples from each muscle that were necessary for a systematic assessment of intra-individual variability.

**Table 1:** Summary of patient and muscle biopsy details. Het., historical homogenate m.3243 A > G heteroplasmy values from post-mortem tissue. CPEO, chronic progressive external ophthalmoplegia; MELAS, Mitochondrial myopathy, Encephalopathy, lLctic Acidosis, and Stroke-like episodes; OMIM, online Mendelian inheritance in man with phenotype # code; QD, *quadriceps femoris*; TA, *tibialis anterior*; M, male; F, female; N, number of biopsies; NA, not available.

Patient	Sex	Age (years) at diagnosis	Age (years) at death	Phenotype # OMIM	Clinical features	*N* biopsies		Het. (%)	
						QD	TA	QD	TA
P1	M	42	61	MELAS syndrome # 540000	Blindness (cortical), dysarthria, **myopathy**, cerebellar ataxia, neuropathy, pyramidal signs, cognitive impairment	5	5	52	NA
P2	M	38	54	MELAS syndrome # 540000	Blindness (cortical), ptosis, CPEO, dysarthria, **myopathy**, cerebellar ataxia, pyramidal signs	4	3	83	85
P3	F	34	53	MELAS syndrome # 540000	Dysarthria, **myopathy**, neuropathy, pyramidal signs	6	5	72	64
P4	F	37	64	MELAS syndrome # 540000	Blindness (cortical), CPEO, dysarthria, **myopathy**, cerebellar ataxia, neuropathy, cognitive impairment	1	2	77	86

### Cryosectioning

**Fig. 1:**
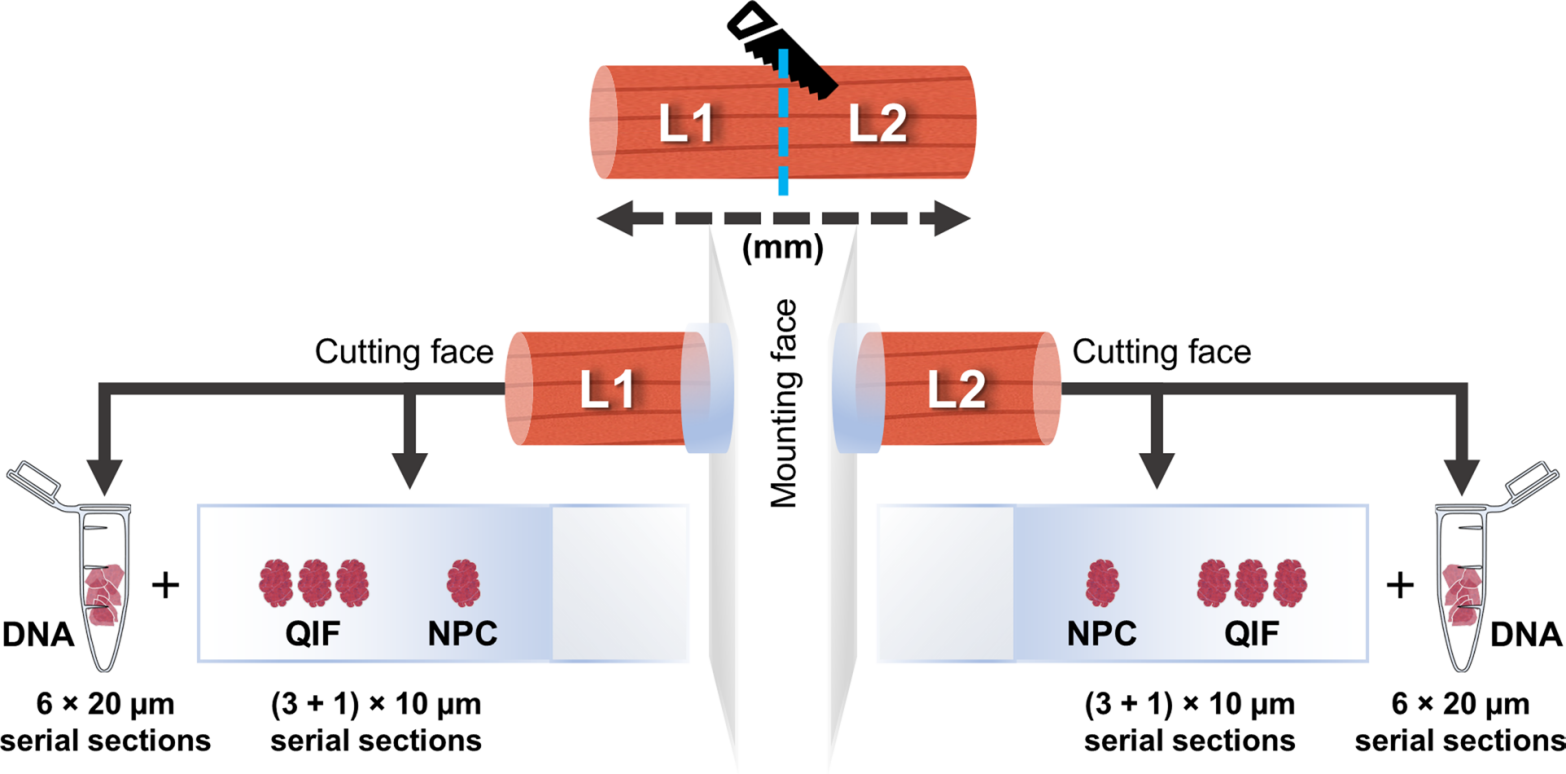
Post-mortem muscle biopsy processing workflow. Example of a larger muscle biopsy that was split transversely across the long (L) axis of fibres into two new biopsy segments, referred to as L-splits (e.g., L1 and L2). From each segment, six 20 μm serial sections were collected for homogenate DNA analysis. This was followed by collection of four 10 μm serial sections onto a glass slide: three grouped for quadruple immunofluorescence (QIF) staining, and a fourth used as a no-primary control (NPC). The original biopsy was measured along its longitudinal axis prior to splitting, and the resulting segments were mounted by their splitting faces, leaving the original extremities exposed for cryosectioning. Consequently, for L-split biopsies, the anatomical distance between sections used for mitochondrial DNA and QIF analyses is known. Smaller biopsies were directly mounted and cut in the same way.

Per biopsy, six 20 μm sections were cut into microfuge tubes for homogenate DNA extraction (Fig. 1). Subsequently, another four 10 μm sections were collected onto one glass slide resulting in triplicate serial sections (S1-S3) for immunofluorescence staining alongside one no-primary control (NPC). If too long for stable mounting for cryo-sectioning (approximately 15 mm), biopsies were split across the fibres’ long (L) axis and the resulting biopsy segments (L-split biopsies) mounted on their split faces for sectioning at the original biopsy’s extremities. Original biopsies were measured so that anatomical distance between sections from L-splits was known. Control tissues were obtained as slides with two sections, including an NPC.

### Immunofluorescent staining, microscopy and image processing

**Fig. 2:**
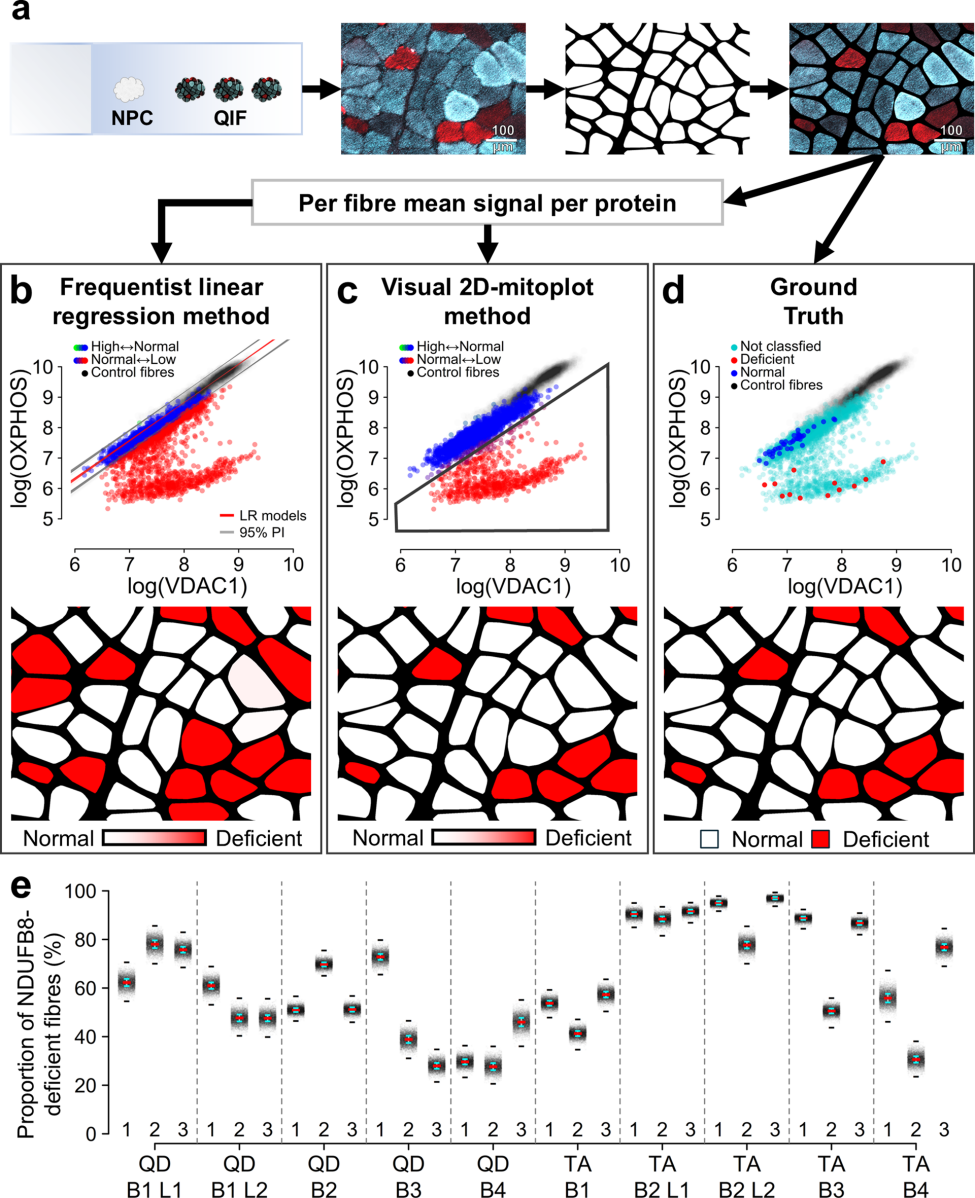
Methods for muscle fibre OXPHOS classification based on quadruple immunofluorescent staining. **(a)** Tissue sections were stained using quadruple immunofluorescence (QIF). Fibres were segmented by intensity thresholding of the membrane marker as previously described^37^. Example microscopy image shows one OXPHOS protein (blue) overlaid with the mitochondrial marker (red, VDAC1); the corresponding segmentation mask is shown in white. Scale bar: 100 μm. Mean per-fibre QIF signal was extracted for classification. **(b) **Frequentist linear regression method: fibres classified using 95% predicted intervals (PI, grey lines) from linear regressions (LR, red lines) fitted to bootstrapped control data (*n* = 10,000 iterations). Colour scale represents per fibre classification certainty as the mean across models. **(c) **Visual 2D-mitoplot method: investigators manually lassoed fibres with relatively high or low OXPHOS signal compared to control-like or ‘normal’ patient fibres. The superimposed polygon outline illustrates how one investigator could visually classify fibres as OXPHOS-deficient. Colour scale represents per fibre classification certainty as the mean label across investigators. Control data was displayed to anchor classifications. **(d) **Ground truth: fibres classified by direct visual inspection of representative ¼ mm² regions from images. **b-d)** In all 2D-mitoplots^24^, fibres are shown in OXPHOS–VDAC1 space: control fibres (black); patient fibres classified for OXPHOS status as high (green), normal (blue), low (red), or not classified (cyan). Under the 2D-mitoplot of each classification effort, OXPHOS-deficient fibres are highlighted on the segmentation mask shown in (**a**), with the colour scale representing classification certainty. **(e)** For both classification methods in (**b**) and (**c**), distributions of proportions of fibres high (overabundant) or low (deficient) in each OXPHOS protein per section were generated by bootstrapping patient fibres. Stripchart shows an example of these per-section distributions for patient 1 according to the frequentist method in (**b**), where each dot (black) represents one bootstrapped value of proportion of NDUFB8-deficient fibres. Dashes are per-distribution median (red), median absolute deviation (cyan) and full range (black).

Sections underwent quadruple immunofluorescence (QIF) as previously described^23^, measuring NDUFB8 (OXPHOS complex I subunit), MT-CO1 (complex IV subunit), VDAC1 (mitochondrial mass marker) and LAMA1 (fibre membrane marker). To minimise technical variability, all sections from each patient were stained in a single batch. For each tissue biopsy, triplicate serial sections (S1-S3) were stained together on the same slide, alongside an NPC (Fig. 2a). Sections were imaged at 20x magnification using a ZEISS Cell Discoverer 7 microscope at Newcastle University’s Bioimaging Unit. Single fibre mean pixel intensity per OXPHOS protein was extracted from images as previously described^37^. In total, QIF of 31 per-biopsy section triplicates yielded 120,306 analysable fibre cross-sections, representing 40,102 unique patient fibres analysed over 30 μm. One section from each of three controls yielded between 1,836 and 4,818 analysable control fibres per batch.

### OXPHOS classification of individual muscle fibres

When classifying fibres for OXPHOS status using the frequentist methodology^24^, the 95% predictive interval of a linear regression model fitted to batch-specific OXPHOS–VDAC1 control data defined the normal OXPHOS range (Fig. 2b). Patient fibres falling below this range were classified as OXPHOS-deficient. Control fibres were bootstrapped with replacement to generate 10,000 linear regression models per OXPHOS protein. Per-fibre classification uncertainty was estimated as the mean classification across all models and patient fibres were classified as deficient if 95% or more of the models concurred.

During fibre classification by visual inspection of OXPHOS–VDAC1 scatter plots, previously described as 2D-mitoplots^24^, three investigators independently identified clusters of OXPHOS-normal patient fibres based on their alignment with control data, relying solely on the plots without reference to microscopy images. Investigators classified fibres under these clusters as OXPHOS-deficient by directly lassoing data points in the plots (Fig. 2c). Per-fibre classification uncertainty was estimated as the mean classification across 10,000 bootstrapped inter-investigator classifications and patient fibres were classified as deficient if 95% or more of the bootstrapped classifications concurred. This method used graphical interfaces created with *Python* v3.9.13 and using the packages *skimage* v0.19.3, *matplotlib* v3.7.0, and *tkinter* v8.6.

The 2D-mitoplot classification method was designed to improve classification reliability while remaining accessible, efficient, scalable and transferable. By abstracting the underlying histology images and providing a simplified set of classification rules, the method does not require the classifying investigator to be familiar with laboratory methods, histological interpretation or detailed disease biology. This abstraction also renders the method largely dataset agnostic, provided that similar classification strategies and assumptions are maintained, particularly the use of an appropriate normalisation marker (e.g., VDAC1) to generate 2D plots in which datapoint clusters can be visually classified using simple rules. Training requirements for investigators are minimal, and each classification event was conservatively estimated to take fewer than 5 s. Importantly, classification time does not increase with the number of datapoints per plot (e.g., fibres) meaning that large data subsets (e.g., tissue sections) can be classified efficiently. A minimum number of datapoints per cluster is nonetheless required for visually recognisable and stable clustering, which will depend on the underlying data distribution. Finally, due to its technical simplicity, the method can be implemented using widely available open-source software and consumer-grade hardware.

### Validation of OXPHOS classification of individual muscle fibres

Visual inspection of microscopy images was considered the ‘ground truth’ for single-fibre OXPHOS status (Fig. 2d). As an exhaustive classification of all images is impractical, labels were derived from a representative ¼ mm² region per section in a subset of 57 sections. This approach yielded ground truth labels for 3,853 fibre segments, corresponding to approximately 5% of fibres in each section.

Bootstrapping patient fibres within each section was used to capture the uncertainty present in the proportion of OXPHOS-deficient fibres per section according to each method (Fig. 2e). As expected, both methods also classified fibres that exceeded the reference range as OXPHOS-overabundant. In keeping with the literature^23,24^, this phenotype was rare (up to 0.03% of all fibre segments according to the visual 2D-mitoplot method), precluding further statistical analysis.

The *R* package *irr* v0.84.1 was used to calculate per-section inter-investigator agreement of visual 2D-mitoplot classifications, using Fleiss’ kappa (*kappam.fleiss*), and to calculate agreement of each classification method with the ground truth, using Cohen’s kappa (*kappa2*). Both classification methods were benchmarked against available ground truth classifications. Whenever appropriate, statistical estimates were weighted by fibre count per section and bootstrapped 1 million times per method and OXPHOS protein to derive means and 95% confidence intervals (CI), comparing classification performance between methods. Benchmarking results are summarised and described in Supplementary Table S1. F1-score summarised methods’ performance as the harmonic mean of Positive Predictive Value (PPV) and Sensitivity: F1-score = 2 × (PPV × Sensitivity)/(PPV + Sensitivity)^39^.

### DNA extraction

Tissue was kept frozen before DNA extraction using QIAamp DNA Micro kits and following QIAGEN’s instructions for genomic DNA isolated from tissue samples under 10 mg. Biopsies from each patient were extracted in the same batch.

### Homogenate m.3243 A > G quantification by pyrosequencing

An amplicon spanning m.3162–3182 was generated by PCR using primers at positions m.3324−3304 and m.3222–3239 (GenBank Accession NC_012920.1). m.3243 A > G heteroplasmy was estimated in triplicate by pyrosequencing, as previously described^40^. As controls, mtDNA with 0% (wild type), 16% (low), 52% (intermediate) and 92% (high) heteroplasmy was measured alongside patient biopsies. Per-biopsy heteroplasmy is reported as the mean of each triplicate.

### Measuring mitochondrial DNA copy number

Mitochondrial DNA copy number (mtDNAcn) was estimated by quantitative PCR (qPCR), as described previously^41^, here using a *B2M*-p7D1 pcDNA3.1^(+)^ plasmid construct (Invitrogen), containing one copy of the target sequences for *MT-ND1* and *B2M*, to generate qPCR standard curves^42^. All samples were measured using six replicates. qPCR is susceptible to target-specific assay variation, which can be magnified when calculating mtDNAcn per nucleus from quantification cycle (C_q_) values (i.e., by normalising mitochondrial *MT-ND1* to nuclear *B2M*). Point-estimate methods that generate qPCR standard curves by fitting linear regression models to dilution replicate means^42^ overlook this critical source of variability. Staged bootstrapping of standard mtDNAcn calculations^42^ was used to capture uncertainty around mtDNAcn values. Standard curve models were first bootstrapped by randomly sampling C_q_ values within each dilution’s replicates, with replacement, across 100,000 iterations. Models were then randomly paired, over 100,000 iterations, with within-biopsy and per-target C_q_ values to calculate *B2M* and *MT-ND1* copy number. These values were used to generate distributions of mtDNAcn per nucleus that capture both the combinatorial uncertainty of *B2M–MT-ND1* pairing and C_q_ variability per biopsy. Results are reported as the median of per-biopsy combinatorial distributions of *MT-ND1/*(*B2M*/2) copies to represent mtDNA copies per nucleus.

### Assessing variability in OXPHOS status and mitochondrial DNA genetics

Intra-individual variability was assessed by generating probabilistic distributions of between-biopsy differences through subtraction of random pairs of values between biopsies within a patient. Pairing biopsies from the same muscle assessed intra-muscle variability, while pairing between different muscles assessed inter-muscle variability. For m.3243 A > G heteroplasmy, replicate values were directly paired between biopsies. Conversely, for OXPHOS deficiency and mtDNAcn, pairing was done using bootstrapped estimates from per-biopsy distributions generated as described herein. Variability in OXPHOS deficiency was also assessed between adjacent sections with pairing at section level for all comparisons. Distributions of absolute differences were used to derive medians with 2.5th − 97.5th percentile values as interpretable estimates of expected variability.

### Statistical analysis

Unless otherwise stated, data analyses and plotting were performed using base *R* v4.5.0 and *Cairo* v1.6.2 for *R*. Equally, images of *in-situ* OXPHOS classifications of QIF images were generated using *Python* v3.9.13 with *PIL (Pillow)* v9.4.0, *NumPy* v1.26.4, *pandas* v1.5.3, *SciPy* v1.10.0, *Shapely* v2.0.5, and *OpenCV* v4.7.0. Whenever appropriate, a *p* value of less than 0.05 was considered statistically significant. No custom algorithms, automation, machine learning, or code packages were developed for this work. Analysis scripts are available from the authors upon reasonable request.

## Results

### Frequentist OXPHOS classification methods often misclassify fibres

**Fig. 3:**
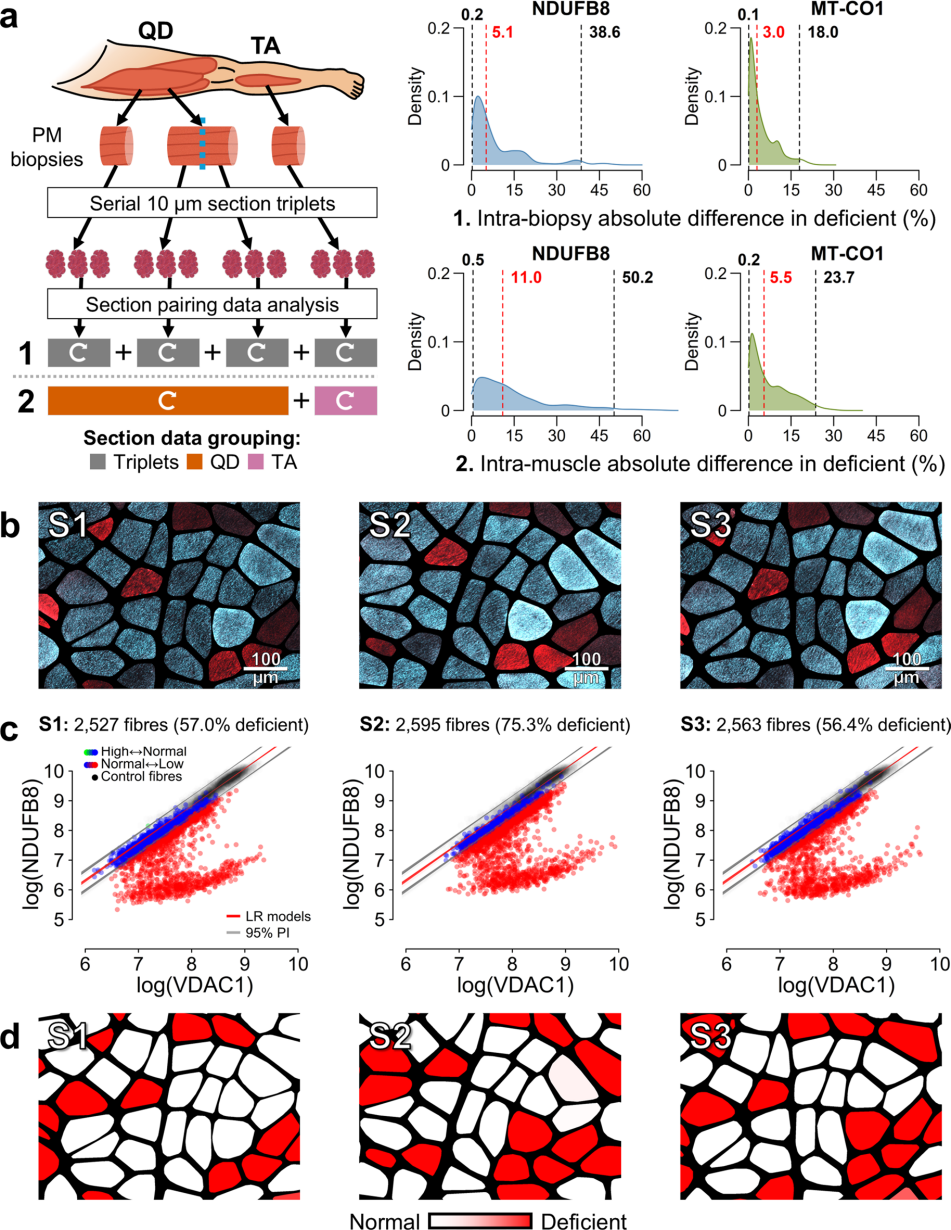
Intra-individual variability in OXPHOS deficiency using the frequentist linear regression classification method. **a)** Schematic overview of tissue sampling and processing from post-mortem (PM) *quadriceps femoris* (QD) and *tibialis anterior* (TA) muscles. OXPHOS variability was estimated by randomly pairing values from per-section distributions of proportions of OXPHOS-deficient fibre (see Fig. 2e) and computing their absolute differences across 1 million iterations. Plots display iteration density (y-axis) of absolute differences (x-axis), with dashed lines indicating the median (red) and 2.5th − 97.5th percentiles (black). All fibre segments included. (**1**) Estimate differences between serial section triplets evaluated per-biopsy inter-replicate variability, independent of muscle or patient. **(2)** Estimate differences between sections from different biopsies within the same muscle and patient evaluated intra-muscle variability. Curved white arrows indicate intra-group pairing. **b)** Example of single-fibre microscopy image cut-outs of equivalent regions across a serial section triplet (S1-S3) displaying overlaid NDUFB8 (blue) and VDAC1 (red) signals. NDUFB8-deficient fibres appear red due to absence of blue signal, while normal fibres appear cyan due to blue–red signal overlay. Scale bar: 100 μm. **c)** NDUFB8 2D-mitoplots^24^ display all analysed fibres corresponding to the section triplet shown in (**b**), illustrating sensitivity of the linear regression classification method to inter-replicate signal variation and patient–control disparity, which reduces classification consistency and precision. Individual fibres are plotted by mean NDUFB8–VDAC1 signal, with a colour scale indicating mean classification across 10,000 bootstrapped models: green = higher than control [+ 1], blue = as control [0], red = lower than control [–1]. Linear regression fits (LR, red lines) and 95% prediction intervals (PI, grey lines) are superimposed. Fibres with mean classification ≤ − 0.95 were considered as deficient to compute the reported per-section proportions of deficiency. **d)** Segmentation masks corresponding to images in (**b**) show deficient fibres highlighted in a colour scale indicating classification certainty across models. These examples illustrate inconsistent classifications across sections and low precision relative to microscopy images.

The frequentist classification method, based on linear regression predictive models, produced median bootstrapped differences in OXPHOS deficiency between serial sections of 5.1% (0.2–38.6: 2.5th − 97.5th percentile) for NDUFB8 and 3.0% (0.1–18.0) for MT-CO1 (Fig. 3a). Inter-biopsy differences within the same muscle and patient were larger, at 11.0% (0.5–50.2) and 5.5% (0.2–23.7), respectively.

Bootstrapped per-section Cohen’s kappa values comparing single-fibre classifications to the ground truth indicated moderate mean agreement, with mean(κ) = 0.569 for NDUFB8 and 0.524 for MT-CO1, weighted by per-section fibre count. Despite negligible False Negative Rates, the bootstrapped weighted mean False Positive Rate for NDUFB8 was 33.2%, with a classification precision of 58.3%, indicating a consistent tendency to overestimate deficiency. For MT-CO1, these values were 7.6% and 43.5%, respectively. Accordingly, the mean weighted F1-score^39^ was 69.9% for NDUFB8 and 58.3% for MT-CO1. Detailed statistics on classification performance are provided in Supplementary Table S1. We identified systematic overall signal disparities between control and patient samples that contribute to recurrent fibre misclassification, while signal variation accounts for inconsistent classifications even between adjacent sections (Fig. 3b–d). Inspection of OXPHOS–VDAC1 scatter plots, also known as 2D-mitoplots^24^, further revealed dense clusters of patient fibres, as illustrated by Fig. 3c, which were confirmed to be OXPHOS-normal in the ground truth dataset; these clusters formed the basis for our improved classification method below.

### The 2D-mitoplot pipeline generated reliable single-fibre classifications leading to consistent estimations of OXPHOS-deficiency across tissues

**Fig. 4:**
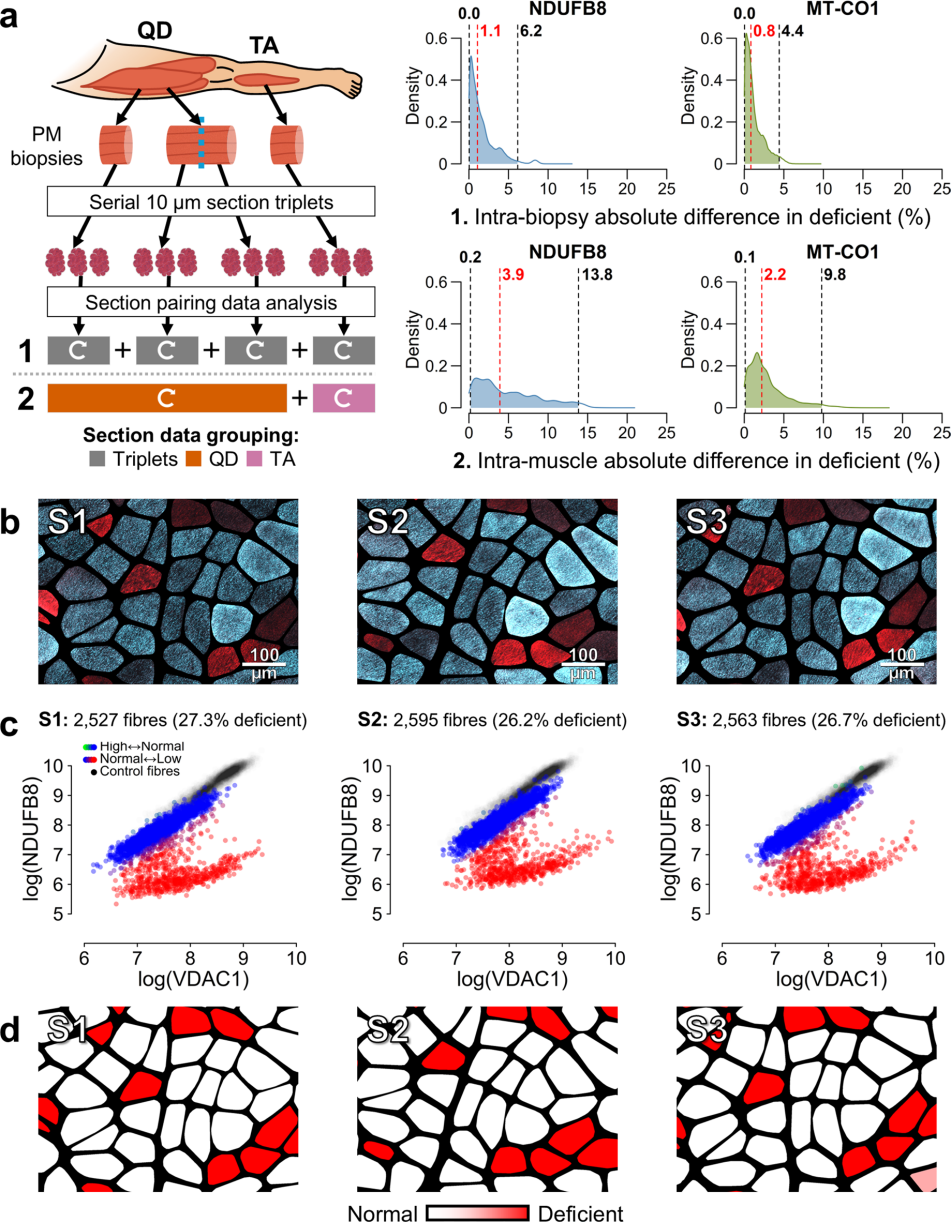
Intra-individual variability in OXPHOS deficiency classification using the visual 2D-mitoplot method. **a)** Schematic overview of tissue sampling and processing from post-mortem (PM) *quadriceps femoris* (QD) and *tibialis anterior* (TA) muscles. OXPHOS variability was estimated by randomly pairing values from per-section distributions of proportions of OXPHOS-deficient fibre and computing their absolute differences across 1 million iterations. Plots display iteration density (y-axis) of absolute differences (x-axis), with dashed lines indicating the median (red) and 2.5th − 97.5th percentiles (black). All fibre segments included. **(1)** Estimate differences between serial section triplets evaluated per-biopsy inter-replicate variability, independent of muscle or patient. (**2**) Estimate differences between sections from different biopsies within the same muscle and patient evaluated intra-muscle variability. Curved white arrows indicate intra-group pairing. **b)** Example of single-fibre microscopy image cut-outs of equivalent regions across a serial section triplet (S1-3) displaying overlaid NDUFB8 (blue) and VDAC1 (red) signals. NDUFB8-deficient fibres appear red due to absence of blue signal, while normal fibres appear cyan due to blue–red signal overlay. Scale bar: 100 μm. **c)** NDUFB8 2D-mitoplots display all analysed fibres from the section triplet shown in (**b**), illustrating the robustness of the visual 2D-mitoplot method, which enables consistent and precise classification of fibres across triplet sections, regardless of inter-replicate signal variation or patient-control disparity. Individual fibres are plotted by mean NDUFB8–VDAC1 signal, with a colour scale indicating their mean classification across 10,000 bootstrapped inter-investigator classifications: green = higher than normal [+ 1], blue = normal [0], red = lower than normal [–1]. Fibres with mean classification ≤ − 0.95 were considered as deficient to compute the reported per-section proportions of deficiency. **d)** Segmentation masks corresponding to images in (**b**) show deficient fibres highlighted in a colour scale indicating classification certainty across bootstrapped investigator classifications. These examples illustrate consistent classification across sections and high precision when compared to microscopy images and the frequentist method in Fig. 3.

In the visual 2D-mitoplot classification method, inter-investigator consistency was confirmed by per-section Fleiss’ kappa statistics, yielding mean(κ) = 0.921 (95% Confidence Interval (CI): 0.907–0.935) for NDUFB8 and 0.810 (95% CI: 0.787–0.833) for MT-CO1, with *p* < 1 × 10^− 16^. Bootstrapped per-section Cohen’s kappa values comparing single-fibre classifications to the ground truth yielded high agreement, with mean(κ) = 0.908 for NDUFB8 and 0.799 for MT-CO1, weighted by per-section fibre count. The bootstrapped weighted mean False Positive Rate was 4.0% for NDUFB8, with a classification precision of 93.4%, higher than the frequentist approach (58.3%). For MT-CO1, these values were 0.6% and 86.7%, respectively. However, although False Negative Rates remained low for NDUFB8, with a mean of 5.5%, this was 20.2% for MT-CO1. This is potentially due to the small numbers of MT-CO1-deficient fibres which affected classification and statistical stability. For instance, while all sections had ≥ 5 NDUFB8-deficient fibres in the ground truth dataset, for MT-CO1 this was only observed in 14/57 sections, with up to 7 sections having insufficient data to complete statistical analysis. Detailed statistics on classification performance are provided in Supplementary Table S1. The visual 2D-mitoplot classification method successfully overcame signal disparities between control and patient samples, and between serial sections, to generate consistent and biologically relevant results (Fig. 4).

Despite offering substantially improved classification reliability over the frequentist method, the 2D-mitoplot approach remained highly efficient. In our study, we conservatively estimated that classifying all 93 sections for both deficiency and overabundance across two proteins took approximately 20 to 25 min per investigator, corresponding to 372 classification events encompassing 120,306 fibres.

### Variability in OXPHOS deficiency increases with anatomical distance between samples

**Fig. 5:**
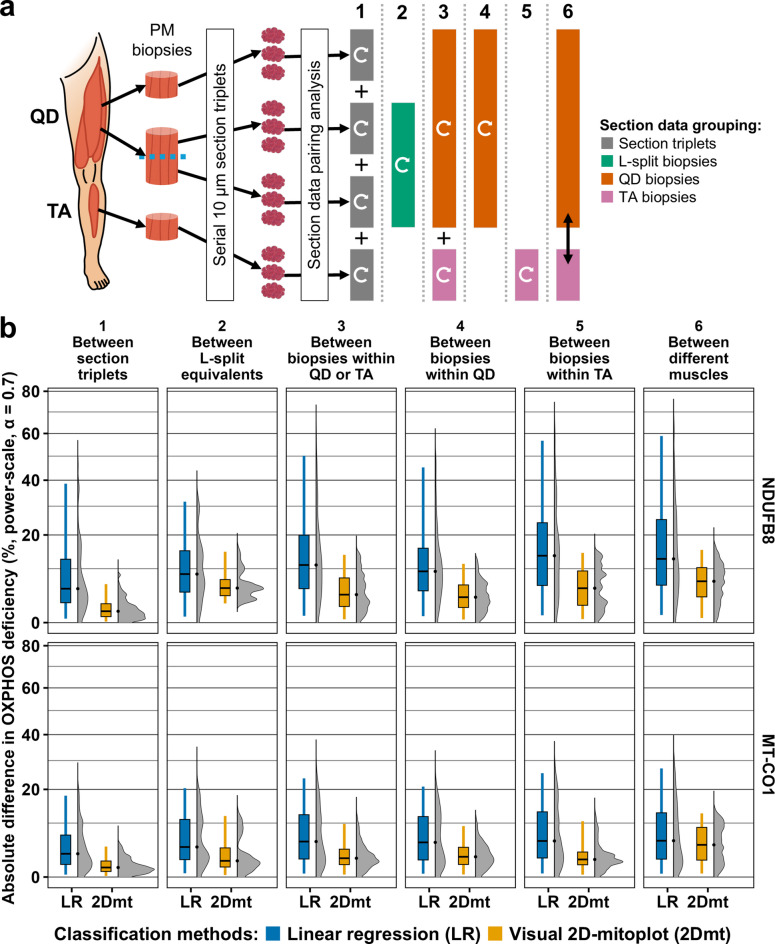
Variability in OXPHOS deficiency with increasing anatomical distance, comparing classification methods. (**a**) Schematic overview of tissue sampling and processing from post-mortem (PM) *quadriceps femoris* (QD) and *tibialis anterior* (TA) muscles. OXPHOS variability was estimated by randomly pairing values from per-section distributions of proportions of OXPHOS-deficient fibre (see Fig. 2e) and computing their absolute differences across 1 million iterations. Six pairing strategies were defined by increasing anatomical distance as follows: **(1) Between section triplets:** involved pairing sections within the same biopsy, with differences pooled across all muscles and patients to estimate intra-biopsy variability; **(2) Between L-split equivalents:** paired sections between corresponding L-split biopsies (i.e., L1 and L2), with differences pooled across all L-split groups to estimate short-range intra-muscle variability; **(3) Between biopsies within QD or TA:** paired sections across different biopsies within the same muscle, with differences from both muscles pooled to estimate overall intra-muscle variability; **(4) Between biopsies within QD** and **(5) Between biopsies within TA:** followed the same approach as (**a3**) but were restricted to QD or TA, respectively; and **(6) Between different muscles: **paired sections between QD and TA to estimate inter-muscle variability. All comparisons were performed within the same patient, with results pooled across patients. Curved white arrows indicate intra-group pairing and the black straight double arrow indicates inter-group pairing. **b)** Distributions of absolute differences in proportions of OXPHOS-deficient fibres organised by OXPHOS protein (rows, right labels), pairing strategies, as described in (**a1–6**), representing increasing anatomical distance (columns, top labels), and per classification method (x-axis labels): Linear regression (LR) in blue and visual 2D-mitoplot (2Dmt) in yellow. Vertical density plots display iteration densities (x-axis) of absolute differences in deficiency (y-axis) showing full range of differences, while box plots show medians and interquartile ranges (IQR) with whiskers representing the 2.5th − 97.5th percentiles. Axis scaling uses x ↦ sign(x) ⋅ ∣x∣^0.7^ for display only; raw values were preserved. Gridlines are spaced at 10% intervals. Patient and biopsy details are provided in Table 1.

In keeping with visual inspection of images, the visual 2D-mitoplot classification method confirmed low variability in the proportion of OXPHOS-deficient fibres between adjacent sections with median bootstrapped differences of 1.1% (0.0–6.2: 2.5th − 97.5th percentile) for NDUFB8 and 0.8% (0.0–4.4) for MT-CO1 (Fig. 4a). Inter-biopsy differences within the same muscle and patient were only slightly higher at 3.9% (0.2–13.8) for NDUFB8 and 2.2% (0.1–9.8) for MT-CO1. Of note, variability across L-split biopsies was modestly higher than between any biopsy, with mean differences of 5.3% (2.3–14.8) for NDUFB8 and 1.8% (0.1–11.9) for MT-CO1 (Fig. 5), despite comparing sections anatomically separated by only 14.4 ± 3.6 mm (mean ± standard deviation). These findings should be interpreted cautiously, as only four pairs of L-split biopsies were included in the dataset, and precise anatomical distances were otherwise unknown. Restricting biopsy pairings to either muscle revealed higher variability in *tibialis anterior* (TA), especially for NDUFB8. Mean differences in *quadriceps femoris* (QD) were 3.4% (0.2–11.3) for NDUFB8 and 2.5% (0.1–9.2) for MT-CO1, while in TA, they were 5.3% (0.2–14.4) and 2.0% (0.1–10.5), respectively. Finally, pairing sections between muscles within a patient led to median differences of 6.8% (0.3–15.3) and 4.8% (0.2–12.7), respectively. MT-CO1 deficiency was substantially lower than NDUFB8 deficiency, averaging 9.4 ± 5.8% (mean ± standard deviation) compared to 29.7 ± 8.7%, across all sections. This could explain the tendency for higher variability for NDUFB8 deficiency, compared to MT-CO1; however, this difference became less marked when comparing sections between muscles. For comparison, these results are summarised in Fig. 5b; Table 2, alongside corresponding variability results according to the frequentist classification method.

**Table 2:** Variability estimates summaries of the variability distributions shown in Fig. 5, estimated by randomly pairing values from per-section distributions of proportions of OXPHOS-deficient fibres (see Fig. 2e) and computing their absolute differences across 1 million iterations. Six pairing strategies were defined by increasing anatomical distance as follows: **(1) Intra-biopsy (between section triplets)**: involved pairing sections within the same biopsy, with differences pooled across all muscles and patients to estimate intra-biopsy variability; **(2) Intra-L-split (between L-split equivalents)**: paired sections between corresponding L-split biopsies (i.e., L1 and L2), with differences pooled across all L-split groups to estimate short-range intra-muscle variability; **(3) Intra-muscle (between biopsies within QD or TA)**: paired sections across different biopsies within the same muscle, with differences from both muscles pooled to estimate overall intra-muscle variability; **(4) Intra-QD (between biopsies within QD)** and **(5) Intra-TA (between biopsies within TA)**: followed the same approach as (**3**) but were restricted to QD or TA, respectively; and **(6) Inter-muscle (between different muscles)**: paired sections between QD and TA to estimate inter-muscle variability. All comparisons were performed within the same patient, with results pooled across patients and presented as percentages. QD, *quadriceps femoris*; TA, *tibialis anterior*; IQR, interquartile range.

NDUFB8	Linear regression classification			Visual 2D-mitoplot classification		
Comparison	Median [IQR] (%)	2.5th − 97.5th centiles (%)	Full range (%)	Median [IQR] (%)	2.5th − 97.5th centiles (%)	Full range (%)
1. Intra-biopsy	5.1 [2.4–12.6]	0.2–38.6	0.0–57.0	1.1 [0.4–2.2]	0.0–6.2	0.0–12.5
2. Intra-L-split	8.6 [4.4–15.1]	0.4–31.7	0.0–44.0	5.3 [3.7–7.2]	2.3–14.8	1.0–20.6
3. Intra-muscle	11.0 [5.2–19.9]	0.5–50.2	0.0–73.5	3.9 [1.8–7.6]	0.2–13.8	0.0–19.8
4. Intra-QD	9.3 [4.7–15.8]	0.5–45.2	0.0–62.3	3.4 [1.6–6.0]	0.2–11.3	0.0–14.3
5. Intra-TA	13.6 [5.9–24.1]	0.6–56.7	0.0–74.8	5.3 [2.0–9.4]	0.2–14.4	0.0–20.4
6. Inter-muscle	12.7 [5.9–25.2]	0.6–58.9	0.0–76.3	6.8 [3.5–10.3]	0.3–15.3	0.0–22.5

MT-CO1	Linear regression classification			Visual 2D-mitoplot classification		
Comparison	Median [IQR] (%)	2.5th − 97.5th centiles (%)	Full range (%)	Median [IQR] (%)	2.5th − 97.5th centiles (%)	Full range (%)
1. Intra-biopsy	3.0 [1.3–7.0]	0.1–18.0	0.0–29.3	0.8 [0.4–1.8]	0.0–4.4	0.0–9.3
2. Intra-L-split	4.3 [2.0–11.0]	0.2–20.4	0.0–35.3	1.8 [0.9–4.1]	0.1–11.9	0.0–18.2
3. Intra-muscle	5.5 [2.1–12.3]	0.2–23.7	0.0–38.1	2.2 [1.2–3.9]	0.1–9.8	0.0–17.6
4. Intra-QD	5.3 [1.9–11.8]	0.2–20.9	0.0–33.8	2.5 [1.2–4.3]	0.1–9.2	0.0–13.7
5. Intra-TA	5.6 [2.3–13.1]	0.2–25.5	0.0–37.4	2.0 [1.2–3.3]	0.1–10.5	0.0–17.3
6. Inter-muscle	5.7 [2.1–12.8]	0.2–27.2	0.0–39.8	4.8 [1.9–8.9]	0.2–12.7	0.0–19.5

### Variability in homogenate mitochondrial DNA copy number modestly increases with anatomical distance between samples

**Fig. 6:**
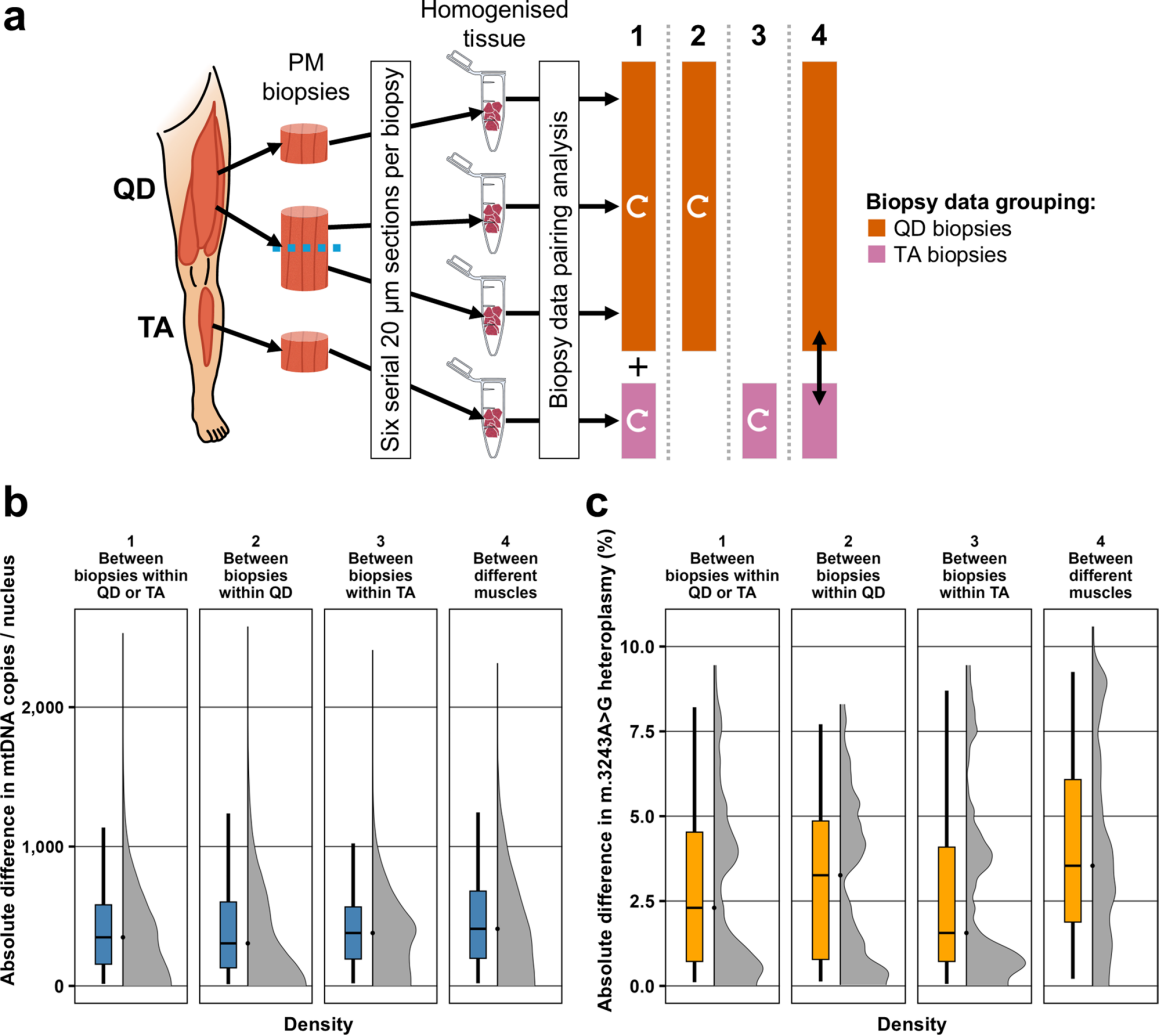
Variability in mitochondrial DNA copy number and m.3243 A > G heteroplasmy with increasing anatomical distance. **a)** Schematic overview of tissue sampling and processing from post-mortem (PM) *quadriceps femoris* (QD) and *tibialis anterior* (TA) muscles. Variability in mtDNA copy number per nuclear genome (mtDNAcn) was estimated by randomly drawing pairs of values from per-biopsy mtDNAcn distributions and computing their absolute differences across 100,000 iterations. The same approach was applied to estimate variability in m.3243 A > G heteroplasmy, based on triplicate pyrosequencing measurements per biopsy. Four pairing strategies were defined by increasing anatomical distance as follows: **(1) Between biopsies within QD or TA:** paired biopsies within the same muscle, with differences from both muscles pooled to estimate overall intra-muscle variability; **(2) Between biopsies within QD** and **(3) Between biopsies within TA:** followed the same approach as (**a1**) but were restricted to QD or TA, respectively; and **6. Between different muscles:** paired biopsies between QD and TA to estimate inter-muscle variability. All comparisons were performed within the same patient, with results pooled across patients. Curved white arrows indicate intra-group pairing and the black straight double arrow indicates inter-group pairing. **b)** Variability in mtDNAcn across the four biopsy pairing strategies (**a1–4**; column labels). Vertical density plots display iteration densities (x-axis) of absolute differences in mtDNAcn (y-axis) showing full range of differences, while box plots show medians and interquartile ranges (IQR) with whiskers representing 2.5th − 97.5th percentiles. **c)** Variability in m.3243 A > G heteroplasmy using the same four biopsy pairing strategies. Density plots and boxplots as in (**b**) but showing iteration densities (x-axis) plotted against absolute differences in m.3243 A > G heteroplasmy (y-axis). Patient and biopsy details are provided in Table 1.

From bootstrapped mtDNA copy number (mtDNAcn) distributions per biopsy, we estimated a mean mtDNAcn per nucleus of 1,476 ± 517 copies (mean ± standard deviation), ranging from 608 to 2,788, across the 31 biopsies from all patients, which is in keeping with previous findings^43,44^. Bootstrapping the differences in mtDNAcn, as previously described for OXPHOS deficiency, resulted in modestly less variability across biopsies of the same muscle and patient, with a median difference of 349 copies (15–1,136: 2.5th − 97.5th percentile), compared to that of pairing biopsies from different muscles, which was 409 (19–1,244) copies (Fig. 6a, b). Therefore, a variability increase linked to anatomical distancing was modest and the observed wide variability ranges were in accordance with the much higher technical noise of qPCR data compared to OXPHOS quadruple immunofluorescence (QIF) or m.3243 A > G pyrosequencing. Restricting biopsy pairings to either muscle showed a narrower variation range for TA, with 380 (19–1,022) copies, but a lower median variability for QD, with 305 (12–1,237) copies.

### Homogenate m.3243 A > G heteroplasmy was mostly stable across biopsies within patients

Within each patient, m.3243 A > G heteroplasmy was consistent between muscles, with a pooled standard deviation of 2.9% across all patients and appearing consistent with historical measurements as shown in Table 1. Whilst the high heteroplasmy estimates observed in P2-4 are in keeping with their Mitochondrial myopathy, Encephalopathy, Lactic Acidosis, and Stroke-like episodes (MELAS) phenotype, P1 exemplifies the well-established weak correlation between m.3243 A > G heteroplasmy and clinical severity^11,45^.

Bootstrapping differences in heteroplasmy between biopsies, like with mtDNAcn, suggests that m.3243 A > G heteroplasmy is slightly less variable within muscle than between muscles within each patient, with estimated median differences of 2.3% (0.1–8.2: 2.5th − 97.5th percentile) and 3.5% (0.2–9.3), respectively (Fig. 6a, c). Therefore, a variability increase linked to anatomical distancing was modest for heteroplasmy despite low technical noise between pyrosequencing replicates. Restricting biopsy pairing to either muscle revealed a narrower variation range for QD, 3.2% (0.1–7.7), but a lower median variability for TA, 1.6% (0.1–8.7).

## Discussion

For decades, skeletal muscle biopsy has remained the gold standard for histochemical and genetic assays for confirmation of mitochondrial disease^22–28^, assessing the progression of pathology in mitochondrial myopathy^22,29–34^, and measuring outcomes in clinical trials^25,35–37^.

Here, we assess intra-individual variation in OXPHOS deficiency, mtDNA copy number (mtDNAcn), and m.3243 A > G heteroplasmy at a single timepoint, both within and between two commonly biopsied muscles, *quadriceps femoris* (QD) and *tibialis anterior* (TA). We provide systematic variability threshold estimates as practical benchmarks for distinguishing genuine chronological change from technical or anatomical variability. We propose guidelines for optimising timepoint re-biopsy strategies and introduce a novel single-fibre OXPHOS classification method to mitigate unwanted sources of variability in these metrics. Together, these efforts aim to enhance the detection of longitudinally meaningful changes and increase confidence that positive findings reflect true time-dependent changes.

Studying per-biopsy serial section replicates led us to conclude that frequentist methods of determining single-fibre OXPHOS status^23,24^ are highly susceptible to deviations in quadruple immunofluorescence (QIF) data, despite best technical practice. This susceptibility led to unreliable classifications and non-biologically determined variability in OXPHOS deficiency across replicates. This variability likely masks genuine biological differences or may be misinterpreted as real chronological change. Along individual fibres, OXPHOS-deficient segments are interspersed with normal regions but do not spatially align across fibres^24,46^, and OXPHOS status switching could not have caused the wide variability observed between intra-biopsy section replicates by the frequentist method (Fig. 3). Although matching control and patient tissues would be best practice to reduce control-patient QIF disparity, our data indicate that additional latent factors substantially undermine the reliability of frequentist classification models based on strict normality thresholds derived from control data. Our visual 2D-mitoplot^24^ manual classification method effectively addresses these challenges by using clusters of patient fibres identified as OXPHOS-normal as internal classification references within the same tissue section (Fig. 2c). This approach successfully minimises latent factors between the reference and abnormal fibre populations, enabling reliable fibre classification aligned with microscopy observations (Fig. 4), and offering an efficient means of analysing large numbers of tissues and fibres.

Importantly, the 2D-mitoplot method does not impose specific tissue size requirements; however, a minimum number of datapoints (e.g., fibres) is needed for the visual recognition of data clusters that underpin the classification logic. We did not encounter sections with insufficient fibre numbers in our dataset but based on our experience with 2D-mitoplot outputs, and assuming all other variables are comparable, we would consider a minimum of around 100 fibres sufficient for confident OXPHOS deficiency classification. This threshold will vary with disease genotype, protein target, and imaging technique, all of which influence cluster density and separation and therefore affect classification performance. The expected prevalence of each fibre phenotype is also relevant, as rarer OXPHOS states, such as overabundant fibres, require larger fibre counts to ensure both reliable classification and adequate sampling for meaningful variability estimates.

Our variability analysis confirms that OXPHOS deficiency varies minimally between replicate sections and increases with greater anatomical separation between compared sections (Fig. 5b). The largest increase occurs between intra-biopsy variability and other intra-muscle variability estimates, with only a modest additional increase seen in inter-muscle variability. The effect of anatomical proximity on variation in mtDNAcn and m.3243 A > G heteroplasmy was even more modest (Fig. 6). In practice, sampling the exact same muscle region across follow-up biopsies is not feasible, and our intra-biopsy comparisons (i.e., section replicates and L-splits) cannot be replicated in real-word longitudinal studies. The minimum feasible anatomical proximity is to compare biopsies within the same muscle, for which we provide variability thresholds essential for interpretation of longitudinal data in m.3243 A > G-related myopathy.

For OXPHOS deficiency using QIF and our reliable visual 2D-mitoplot classification method, we propose that, when comparing biopsies within the same muscle, only absolute timepoint differences exceeding 13.8% for NDUFB8 and 9.8% for MT-CO1 may be interpreted as time-dependent, as these values exceed the 97.5th percentile of the observed variability. Additionally, due to its size and modestly lower variability in our data, QD may be a more suitable candidate than TA for QIF OXPHOS studies involving repeated biopsies (Table 2).

Homogenate mtDNAcn varied substantially across biopsies, likely reflecting its known high technical variability^43,44^, as well as the presence of mtDNAcn extremes observed in our data both within and between muscles. We estimate that, when comparing biopsies within the same muscle, only absolute timepoint differences in mtDNAcn exceeding 1,136 copies per nuclear genome may be interpreted as time-dependent, as they surpass the 97.5th percentile of observed mtDNAcn variability (Fig. 6b). This threshold was lower within TA than QD (1,022 vs. 1,237 copies), with that of QD only marginally below the threshold of 1,244 copies observed for inter-muscle comparison. However, median differences indicate that QD was overall less variable than TA (305 vs. 380 copies). In contrast, m.3243 A > G heteroplasmy levels were consistent across replicates and biopsies, showing low variation even between different muscles (Fig. 6c). We estimated that only absolute differences exceeding 8.2% between biopsies within the same muscle may be interpreted as time-dependent changes, as they surpass the 97.5th percentile of observed m.3243 A > G heteroplasmy variability. Here, the threshold was lower within QD than TA (7.7 vs. 8.7%), with that of TA only marginally below the 9.3% threshold for inter-muscle comparisons. However, median differences indicate that TA was overall less variable than QD (1.6 vs. 3.2%).

Optimally, anatomical proximity between follow-up biopsies should be prioritised, and independent experimental replicates (e.g., serial sections) are recommended to confirm technical and classification consistency. With patient comfort and safety in mind, time between biopsies should allow sufficient healing to ensure that obtaining follow-up samples from the same area becomes ethically and scientifically acceptable. However, it is important to note that the overall increase in variability between intra-muscle and inter-muscle comparisons was modest for OXPHOS deficiency and marginal for mtDNA experiments. Therefore, if short follow-up intervals are unavoidable, increasing the anatomical distance between biopsy sites to avoid regenerative changes is not expected to substantially introduce additional spatial variability. Previous interventional studies using similar mitochondrial assays to assess the effect of exercising in muscle mitochondrial function in other myopathic conditions, have reported good results with follow-up biopsies taken at least 2 cm apart and after a minimum interval of 12 weeks^25,37^. In our study, differences in variability between TA and QD were also modest and inconsistent across assays, not strongly supporting the choice of either muscle. Biopsy strategies should instead be guided by other experimental requirements, considering that QD is a larger muscle ideal for repeated biopsies, while TA biopsy procedures are less invasive.

Altogether, our findings further provide valuable insights into m.3243 A > G-related myopathy suggesting that real-world variability in OXPHOS dysfunction, mtDNAcn and heteroplasmy tends to be relatively similar within muscle tissues and at least between QD and TA. To our knowledge, no other studies have comprehensively assessed intra-individual variability in widely used genetic and histochemical assays for mitochondrial disease and dysfunction. Most published data are either restricted to small series or case reports^33,34^, or involved limited sampling or assays not comparable to ours^25,36,47–49^, precluding meaningful direct comparisons with our data. Noticeably, one study using the same QIF OXPHOS assay reported a reduction in the proportion of OXPHOS-deficient fibres following 12 weeks of resistance exercise training in individuals with myotonic dystrophy type 1^37^. Biopsies were obtained from the same QD but intentionally spaced ≥ 2 cm apart to avoid regenerative changes induced by the first biopsy. Nonetheless, even studies that considered sound re-biopsy strategies^25,37^ paid limited attention to the impact of intra-muscle variability and employed insufficient experimental replicates to assess methodological reliability or define tolerance thresholds for interpreting findings.

Evidence suggests that QIF against NDUFB8 and MT-CO1 correlates well with both traditional COX/SDH histochemical assays^23^ and modern, spatially resolved, antibody-based assays, such as imaging mass cytometry^24^, when assessing OXPHOS function in skeletal muscle. Likewise, fibres’ OXPHOS status based on these protein markers often correlates with their status for other OXPHOS proteins, particularly within the same complex^24^. Yet, these studies revealed that this is not always the case and that the threshold for deficiency^20^ can be specific for genotype, OXPHOS complex and even protein^23,24^. Therefore, while our results provide valuable insights for inferring thresholds to interpret existing and future data, they are limited by our small patient cohort and only directly applicable to studies on m.3243 A > G using equivalent targets and methodologies, and are not directly applicable to other diseases, techniques or molecular markers. Ideally, similar studies should be repeated across a range of diseases that share comparable mitochondrial pathology, using equivalent assays across a range of molecular markers to define appropriate disease-, tissue-, assay- and marker-specific threshold values. Similar conclusions may be drawn from our genetic data; however, these are further limited by our use of tissue homogenate. In future studies, single-fibre approaches should be employed to better capture the mosaic pattern of muscle pathology in these conditions. Additionally, homogenate assays unavoidably include other cell types besides fibres, which may particularly skew mtDNAcn per nucleus measurements, an issue avoided by single-fibre approaches. However, although our study focused on muscle pathology in mitochondrial myopathy, the methodology is tissue agnostic and could be applied to tissues that are more relevant for investigating non-myopathic mitochondrial phenotypes.

While we were able to obtain multiple samples from each patient’s muscle, yielding a substantial number of fibres from a diverse set of sections and enabling robust estimation of intra-individual variability, identifying cases with sufficient material of adequate quality proved challenging. As a result, our study includes a limited number of cases. Larger cohorts will be required to more comprehensively characterise intra-individual variability across target patient populations and to increase confidence in the generalisability of these thresholds.

Although our methodologies could be validated to increase precision of diagnostic studies of muscle biopsies, due to the widespread availability of fast, cost-effective genetic testing using minimally invasive samples (e.g., blood, urine or buccal swab), muscle biopsy is now a rare diagnostic requirement, even in mitochondrial myopathy^50^. However, clinical prognostication remains a challenge in mitochondrial myopathies in part due to methodological unreliability and lack of reference data. Our work directly addresses these limitations by providing a methodology to both develop and apply benchmarks for interpreting natural variability and longitudinal change in clinical studies elucidating disease heterogeneity and mechanisms, validating non-invasive biomarkers, or testing therapies, all using OXPHOS, mtDNAcn or variant heteroplasmy as reference measures of target-tissue pathology. Therefore, our work has the potential to support the development of more robust disease models, stratification and prognostication tools, as well as the implementation of new treatments.

In conclusion, we demonstrate that traditional frequentist OXPHOS classification methods are unreliable due to assay- or spatial-dependent variability and propose our validated 2D-mitoplot method as a reliable, efficient, and scalable alternative that requires minimal resources and expertise. Provided that the data structure, classification logic, and overarching objectives remain similar, our method is agnostic to the source of data, including laboratory technique, tissue type, disease context, or spatial unit, making it readily adaptable for datapoint classification across a wide range of experimental settings. We also demonstrate that longitudinal studies should prioritise spatial proximity of follow-up biopsies to mitigate spatial-dependent variability, increasing the chances of detecting meaningful time-dependent changes. However, our data also suggests that intra-individual variability in m.3243 A > G-related muscle pathology is relatively homogeneous across skeletal muscle tissues, in keeping with the relatively generalised myopathic phenotype of patients. We provide the most detailed characterisation of variability in widely used assays measuring OXPHOS-deficiency, mtDNAcn and m.3243 A > G heteroplasmy in skeletal muscle and propose tolerance thresholds to aid in interpretation of these results in clinical studies into disease progression, ageing or clinical trials. Nevertheless, we acknowledge the limitations of our work, particularly the small number of cases and the inherent challenges of establishing comprehensive reference values in mitochondrial diseases due to their remarkable genotype-phenotype heterogeneity and rarity, especially when considering individual clinical entities. Consequently, a more comprehensive understanding of the expected natural variability of molecular markers will require the replication of similar studies on larger cohorts across a broad spectrum of mitochondrial myopathies.

## Supplementary Information

Below is the link to the electronic supplementary material.

Supplementary Material 1

## Data Availability

The data that support the findings of this study are available upon reasonable request and if in accordance with the respective research ethics boards policies.
