# Supplementary material for: Quantifying variability of mitochondrial markers in m3243A > G myopathy
Source: Sci Rep. 2025 Dec 19;16:3191. doi: 10.1038/s41598-025-33106-3 (PMC12830596; doi:10.1038/s41598-025-33106-3)
Supplement: Supplementary file 1 — Supplementary Material 1 [file 41598_2025_33106_MOESM1_ESM.docx]

**SUPPLEMENTARY INFORMATION**

**Quantifying Variability of Mitochondrial Markers in m3243A>G Myopathy**

Tiago M. Bernardino Gomes, Jordan B. Childs, Valeria Di Leo, Charlotte Warren, Gavin Hudson, Doug M. Turnbull, Conor Lawless, Amy E. Vincent

| **NDUFB8** | **Linear regression classification** | **Visual 2D-mitoplot classification** |
| --- | --- | --- |
| **Metrics** | **mean (95% CI)** | **mean (95% CI)** |
| **Cohen's *kappa*** | 0.569 (0.488–0.643) | 0.908 (0.892–0.925) |
| **Cohen's *z* score** | 5.774 (4.708–6.857) | 8.161 (7.393–8.971) |
| **Cohen's *p*-value** | 0.066 (0.029–0.112) | < 1×10-16 |
| **Sensitivity** | 0.994 (0.986–1) | 0.945 (0.921–0.966) |
| **Specificity** | 0.668 (0.584–0.743) | 0.96 (0.943–0.976) |
| **FNR** | 0.006 (0–0.014) | 0.055 (0.034–0.079) |
| **FPR** | 0.332 (0.257–0.416) | 0.04 (0.024–0.057) |
| **PPV** | 0.583 (0.514–0.648) | 0.934 (0.915–0.953) |
| **NPV** | 0.997 (0.992–1) | 0.989 (0.984–0.994) |
| **Balanced accuracy** | 0.831 (0.79–0.868) | 0.953 (0.942–0.963) |
| **F1-score** | 0.699 (0.636–0.756) | 0.934 (0.924–0.945) |
| **MT-CO1** | **Linear regression classification** | **Visual 2D-mitoplot classification** |
| **Metrics** | **mean (95% CI)** | **mean (95% CI)** |
| **Cohen's *kappa*** | 0.524 (0.443–0.603) | 0.799 (0.724–0.868) |
| **Cohen's *z* score** | 5.318 (4.527–6.149) | 7.443 (6.287–8.629) |
| **Cohen's *p*-value** | 0.038 (0.004–0.089) | 0.017 (0–0.056) |
| **Sensitivity** | 1 (1–1) | 0.798 (0.709–0.881) |
| **Specificity** | 0.923 (0.898–0.945) | 0.993 (0.989–0.996) |
| **FNR** | 0 (0–0) | 0.202 (0.12–0.291) |
| **FPR** | 0.076 (0.055–0.099) | 0.006 (0.003–0.01) |
| **PPV** | 0.435 (0.353–0.52) | 0.867 (0.793–0.931) |
| **NPV** | 1 (1–1) | 0.992 (0.988–0.996) |
| **Balanced accuracy** | 0.962 (0.949–0.973) | 0.895 (0.851–0.937) |
| **F1-score** | 0.583 (0.499–0.665) | 0.827 (0.759–0.889) |

Supplementary Table S1. Summary of distributions of per-section bootstrapped benchmarking statistics comparing each OXPHOS classification method with the ground truth at the single-fibre level, with bootstrapped summary estimates weighted by fibre count per section whenever appropriate. These include means and 95% confidence intervals (CI) per benchmark metric for each method and per OXPHOS protein. FNR, False Negative Rate; FPR, False Positive Rate; PPV, Positive Predictive Value; and NPV, Negative Predictive Value. F1-score summarises methods’ performance as the harmonic mean of Positive Predictive Value (PPV) and Sensitivity: F1-score = 2 × (PPV × Sensitivity) / (PPV + Sensitivity). F1-score reference: Christen, P., Hand, D. J. & Kirielle, N. A Review of the F-Measure: Its History, Properties, Criticism, and Alternatives. *ACM Comput. Surv.* 56, 1-24 (2023).
